# A novel method for culturing enteric neurons generates neurospheres containing functional myenteric neuronal subtypes

**DOI:** 10.1016/j.jneumeth.2024.110144

**Published:** 2024-07

**Authors:** Arabinda Mandal, Chioma Moneme, Bhanu P. Tewari, Allan M. Goldstein, Harald Sontheimer, Lily Cheng, Sean R. Moore, Daniel Levin

**Affiliations:** a Department of Surgery, University of Virginia, Charlottesville, VA, USA; b Department of Neuroscience, University of Virginia, Charlottesville, VA, USA; c Department of Pediatric Surgery, Massachusetts General Hospital, Boston, MA, USA; d Department of Pediatrics, Division of Pediatric Gastroenterology Hepatology, and Nutrition, University of Virginia, Charlottesville, VA, USA

**Keywords:** Enteric nervous system (ENS), Longitudinal muscle myenteric plexus (LMMP), Neurosphere, Enteric neurons, Neuron subtypes, Myenteric plexus, Neural progenitor cells, Neural stem cells, Action potential

## Abstract

**Background:**

The enteric nervous system (ENS) is comprised of neurons, glia, and neural progenitor cells that regulate essential gastrointestinal functions. Advances in high-efficiency enteric neuron culture would facilitate discoveries surrounding ENS regulatory processes, pathophysiology, and therapeutics.

**New method:**

Development of a simple, robust, one-step method to culture murine enteric neurospheres in a 3D matrix that supports neural growth and differentiation.

**Results:**

Myenteric plexus cells isolated from the entire length of adult murine small intestine formed ≥3000 neurospheres within 7 days. Matrigel-embedded neurospheres exhibited abundant neural stem and progenitor cells expressing Sox2, Sox10 and Msi1 by day 4. By day 5, neural progenitor cell marker Nestin appeared in the periphery of neurospheres prior to differentiation. Neurospheres produced extensive neurons and neurites, confirmed by Tubulin beta III, PGP9.5, HuD/C, and NeuN immunofluorescence, including neural subtypes Calretinin, ChAT, and nNOS following 8 days of differentiation. Individual neurons within and external to neurospheres generated depolarization induced action potentials which were inhibited in the presence of sodium channel blocker, Tetrodotoxin. Differentiated neurospheres also contained a limited number of glia and endothelial cells.

**Comparison with existing methods:**

This novel one-step neurosphere growth and differentiation culture system, in 3D format (in the presence of GDNF, EGF, and FGF2), allows for ∼2-fold increase in neurosphere count in the derivation of enteric neurons with measurable action potentials.

**Conclusion:**

Our method describes a novel, robust 3D culture of electrophysiologically active enteric neurons from adult myenteric neural stem and progenitor cells.

## Highlights

• Novel method of single step enteric neurosphere and differentiated neuron culture.
• Isolation method of entire LMMP from small intestine.
• High yield of neurosphere counts.
• Neurospheres differentiate predominantly into neuron and neuronal subtypes.
• Individual neurosphere neurons generate depolarization induced action potentials.

## 1 Introduction

The enteric nervous system (ENS) is comprised of a vast neural network in the gastrointestinal tract responsible for a wide array of homeostatic regulatory functions including gastrointestinal secretion, regulation of motility, absorption of nutrients, sensation of stimuli, and blood flow (Lomax et al., 2005; Furness, 2012; Fleming II et al., 2020). Enteric neurons occur in groups of ganglia interconnected by bundles of nerve fibers to form two main ganglionated plexuses, namely the myenteric plexus between the longitudinal and circular muscle layers, and the submucosal plexus under the circular muscle layer (Osorio et al., 2011). The ENS interconnected network is composed of enteric neurons, glia and neural progenitor cells coordinating adaptation to microenvironmental changes (Bondurand et al., 2003, Schäfer et al., 2009; Gulbransen et al., 2012).

In addition to its regulatory effect, the importance of ENS is emphasized by the life-threatening effects of certain enteric neuropathies like congenital Hirschsprung disease, acquired Chagas disease, diabetic neuropathy, opioid-induced bowel dysfunction, postoperative ileus, inflammatory bowel disease or acute appendicitis (Xiong et al., 2000, Villanacci et al., 2008; Bagyanszki et al., 2012; Furness, 2012). Accumulating evidence suggests that debilitating neurodegenerative conditions like Parkinson’s disease are associated with neuronal damage not only in the brain but also in the ENS (Liddle et al., 2018; McQuade et al., 2021). Like neurons, the non-neuronal glial cells of the ENS also play an essential role in maintaining the integrity of the gastrointestinal tract as its dysfunction leads to inflammatory neuronal degeneration and changes in neurotransmitter expression (Bush et al., 1998, Cornet et al., 2001; Aube et al., 2006). Enteric glial cells are known to produce immunoregulatory molecules that regulate tissue repair and host defence (Progatzky et al., 2022). Clearly, the ENS is essential for normal gastrointestinal function and its pathophysiology (Spencer et al., 2020). So, a thorough understanding of the ENS is critical for elucidating its normal functional influence and the pathophysiology of the gastrointestinal tract including the side effects from pharmacological agents. Studies of enteric neurons in culture are effective models to study the pathophysiology of these enteric neuropathies.

To date only a very limited number of mouse enteric neuron culture methods are available (Smith et al., 2013, Grundmann et al., 2015; Wahba et al., 2016; Brun et al., 2018; Zhang et al., 2021), however mostly with limited yields. It is known that self-renewing multipotent ENS progenitor cells (ENSPC) are able to generate neurons and glia derived from neural crest stem cells. These ENSPCs can be harvested from the neonatal and adult gut (Kruger et al., 2002, Bondurand et al., 2003). Enteric neural stem cells has been isolated through the generation of neurospheres (Bondurand et al., 2003; Schafer et al., 2003; Almond et al., 2007). Neurospheres are aggregates of neural stem and progenitor cells that differentiate into neurons and glial cells when cultured in vitro. This allows both self-renewal and expansion of precursor cells to form cellular aggregates (Reynolds et al., 1996; Schafer et al., 2003; Almond et al., 2007).

In the present study, we describe the isolation of the myenteric plexus from adult mice to form neurospheres in 3D culture in the presence of EGF, FGF2, and GDNF growth factors. In a single step culture, these neurospheres are subsequently differentiated into neuronal subtypes that are capable of generating action potentials. The differentiated neurospheres predominantly express neurons but also contain limited glia and endothelial cells.

## 2 Materials and methods

### 2.1 Isolation of longitudinal muscle myenteric plexus

C57BL/6 J mice aged 7–22 weeks were used in accordance with institutional Animal Care and Use Committee protocols. Animal were sacrificed using CO_2_ inhalation (4 liters per minute, 2 min) followed by cervical dislocation and the abdomen of the mice were thoroughly sprayed with 70% ethanol. A longitudinal incision was made to open the abdomen. The small intestine from the ileum to duodenum was collected while removing the mesentery and placed in a 10 cm petri dish containing ∼10 ml of DPBS with Pen-Strep (1x) on ice. Using gavage needle (size 20), 20 ml of DPBS/Pen-Strep (1x) solution (Supplemental Table) was used to wash the small bowel lumen from proximal to distal end while pulling the intestine onto the needle, x2. The cleaned small bowel was then placed in a 9 cm black silicone coated dissection petri dish with ∼40 ml of cold DPBS/Pen-Strep (wash) solution.

**Fig. 1:**
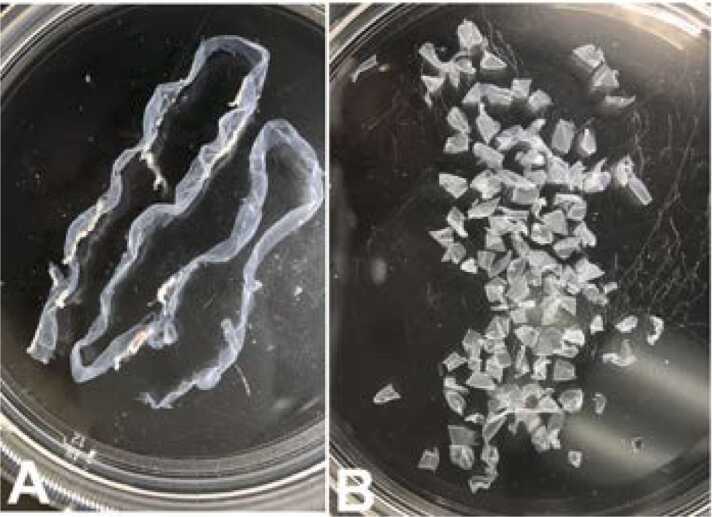
Isolation of LMMP from small intestine. A: LMMP isolate from duodenum, jejunum & ileum from one mouse; B: LMMP, ∼3–5 mm pieces without mesenteric fat from two small intestines.

Next, using forceps and scissors open the bowel along the mesenteric line from duodenum to ileum. Then flatten the small intestine entirely using a bent forceps by gently patting the outer wall of the small intestine keeping the lumen side down using the pins to hold the specimen taut under the wash solution. The LMMP was isolated as described previously (Levin et al., 2020). Using a sterile cotton tipped applicator dipped in cold DPBS/Pen-Strep a LMMP window was created at the ileum end by gently rubbing longitudinally under a dissection stereo microscope. After creating a floating LMMP layer, rearrange the pins as needed for counter pressure and use a wet cotton tipped applicator to gently pull the LMMP segment gradually from the entire length of the small intestine. The average time to isolate the whole LMMP from a clean flattened small intestine was about 20–25 min. The LMMP was then washed once in ∼20 ml of HBSS containing calcium, magnesium and Pen/Strep, x1 and then cut into 3–5 mm pieces, trim any remaining mesenteric fats using a sterile blade and forceps, then store in HBSS/Pen-Strep on ice until used for enzymatic digestion (Fig. 1A, B).

### 2.2 LMMP immunofluorescence

The LMMP samples were spread with pins on silicone coated black petri dishes in DPBS with Pen/Strep and immunostained at room temperature as described earlier (Levin et al., 2020). Briefly, samples were fixed in 5 ml of 4% PFA in PBS for 30 min, then washed x3 in 10 ml of DPBS and then blocked and permeabilized for 60 min in 5 ml of 5% normal goat serum and 0.1% Triton X-100 in DPBS. The LMMP was then incubated with 5 ml of blocking permeabilization (BPB) buffer containing TUBB3 or mouse IgG2a isotype control antibody at 5 µg/ml for 3 h and then washed x3 in 10 ml DPBS. The samples were then incubated with goat anti-mouse IgG F(ab')_2_ -AF488 at 2 µg/ml in BPB for 1 h and washed x3 in 10 ml of DPBS. The samples were further washed in 10 ml of distilled water x2, mounted in Slow Fade diamond antifade DAPI on concave slides, edges sealed using clear acrylic nail polish and imaged using widefield Olympus BX51 fluorescence microscope.

### 2.3 Neurosphere, neuron growth (NSNG) and differentiation (NSND) media

The neurosphere neuron growth media was prepared in 50 ml aliquots in BSL II hood. To 45.8 ml of advanced DMEM/F12 media, add 0.5 ml Pen-Strep 100x, 1.0 ml B27 supplement 50x, 0.5 ml N2 supplement 100x, 0.5 ml Glutamax 200 mM, 0.5 ml HEPES 1 M, 1.0 ml Fetal Bovine Serum (heat inactivated), 50 μL epidermal growth factor (EGF, 20 μg/ml DPBS), 50 μL FGF2 (bFGF, basic fibroblast growth factor, 20 μg/ml DPBS in 0.1% BSA), 50 μL GDNF (glial cell line-derived neurotrophic factor, 25 μg/ml DPBS in 0.1% BSA), and 50 μL Heparin, 0.2%. The mix was filtered through a sterile 0.22 μm Steriflip filter. The growth factors were added only before adding to the cells.

The neurosphere neuron differentiation media is same as the neurosphere growth media but without EGF, FGF2 and Heparin.

### 2.4 Enzymatic digestion of LMMP and 3D neurosphere culture and differentiation

To generate neurospheres, LMMP was digested to produce myenteric plexus as described by Grundmann et. al., (2015) with modifications. Immediately after obtaining the LMMP pieces from one small intestine, the strips were transferred with forceps equally to 2 wells of a 12 well tissue culture plate (not in 1.5 ml tubes, Grundmann et. al., 2015) containing 830 μL of HBSS with calcium, magnesium and Pen/Strep 1x, 150 μL Liberase TH to a final concentration of 0.75 mg/ml, and 20 μL DNase I and allowed the digestion for 4 h at 37 °C in a rocking water bath at 30 rpm. The digested LMMP pieces from each well were then gently washed (without the very low amplitude shaking procedure, Grundmann et. al., 2015) in 5 ml of HBSS with calcium, magnesium and Pen/Strep in a 6 well plate using 1 ml pipet tip for 3 times to remove the digested cells without breaking the myenteric plexus pieces. All LMMP pieces were then pooled into a 50 ml tube containing 2 ml of NSND media, centrifuged at 200xg, 2 min at room temperature (RT). The pellet was mixed aggressively with 100 µl of NSNG using a sterile gel loading tip (bore 0.5 mm) for 20 times and then mixed with 2 ml of NSNG media. The mix was then passed through a 70 µm cell strainer and then again through a 37 µm cell strainer. Spin the filtrate at 300xg, 3 min at RT. The pellet (barely visible) was suspended in 1350 µl of ice cold matrigel and collagen I mix (4:1) by ∼20 trituration using 200 µl and 1000 µl pipet tips. A 100 µl mix was spread in 35 mm MatTek glass bottom dishes with 14 mm micro well. A total of 12 dishes were plated from one small intestine LMMP samples. The dishes were warmed at 37 °C for 11 min and incubated with 3 ml of NSNG media at 37 °C and 5 % CO_2_. The media was changed every 48 or 72 h. To initiate differentiation of the neurospheres, the NSNG media was replaced with NSND media after 5–7 days in culture. Neurospheres count, growth, and differentiation were imaged using EVOS M7000 microscope (ThermoFisher Scientific).

### 2.5 Propidium iodide staining of 4 h digested LMMP

After 4 h digestion and 3 washes in HBSS, only a few LMMP samples were placed in 5 ml of HBSS with Ca^++^, Mg^++^ and Pen/Strep containing 2 µg/ml of propidium iodide and incubated at 37 °C for 10 min (modified after Grundmann et al., 2015, 50 ng/ml; Malik et al., 2015, 20 µg/ml; Levin et al., 2020, 1.0 µg/ml). The treated LMMPs were then washed in 5 ml of HBSS for 3 times, then immediately fixed in 4% PFA in PBS at RT for 10 min. The fixed samples were then permeabilized in 0.5% Triton X-100 in 5 ml HBSS for 15 min at 37 °C, then washed in 5 ml HBSS for 3 times, then mounted in concave slides containing Slow Fade DAPI, and sealed with clear nail polish. For total cells, 0 h digested LMMP samples were permeabilized, washed, fixed, washed, stain with propidium iodide, washed, mounted and sealed as above. Confocal z-stack images were taken in Zeiss LSM 880 microscope.

### 2.6 Immunofluorescence of neurospheres, differentiated cells and quantitation of fluorescence area

Neurospheres and differentiated cells embedded in the matrigel-collagen matrix were fixed in 3 ml of 4% PFA in PBS immediately after removing the culture media for 30 min at 37 °C, then washed with 5 ml of warm DPBS for 3 times at RT. The samples were then blocked and permeabilized with 2.5 ml of 5% normal goat serum and 0.2% Triton X-100 (blocking buffer) for 60 min in DPBS at room temperature. The permeabilized cells were then incubated in primary (in mouse or rabbit) or isotype control antibody at 5 µg/ml in 2.5 ml of blocking buffer, for overnight at RT. The cells were then washed in 5 ml of DPBS at 37 °C DPBS for 3 times followed by incubation with the secondary antibody (goat anti-mouse or anti-rabbit IgG F(ab)_2_ AF488/AF594) at 2 µg/ml in the blocking buffer for 60 min at RT. The cells were then washed in 5 ml of warm DPBS for 3 times, covered with few drops of Slow Fade DAPI, the glass bottom coverslip was then mounted on a concave slide, removed excess DAPI, and sealed with clear acrylic nail polish. Confocal z-stack images were taken in Zeiss LSM 880 microscope with AiryScan. To quantify the total fluorescence area, the maximum intensity projection fluorescence images were analyzed using a hybrid cell count software with BZ-H4C analytic application (Seki et al., (2021); https://www.keyence.com/products/microscope/fluorescence-microscope/bz-x700/models/bz-h4c/.

### 2.7 Action potential generation

The cells were identified under an upright microscope (Leica DMLFSA) with ×40 water immersion lens and infrared illumination. Whole-cell current-clamp recordings were achieved using an Axopatch 200B amplifier (Molecular Devices) as previously described (Tewari et al., 2018). Patch pipettes of 6–8 MΩ open-tip resistance were created from standard borosilicate capillaries (WPI, 4IN THINWALL Gl 1.5 OD/1.12 ID) using PMP 102 pipette puller from Warner Instruments. Patch pipettes were filled with an intracellular solution of 134 mM potassium gluconate, 1 mM KCl, 10 mM 4-(2-hydroxyethyl)-1-piperazineethanesulfonic acid (HEPES), 2 mM adenosine 5′-triphosphate magnesium salt (Mg-ATP), 0.2 mM guanosine 5′-triphosphate sodium salt (Na-GTP) and 0.5 mM ethylene glycol tetraacetic acid (EGTA) (pH 7.4, 290–295 mOsm). Unless otherwise stated, we added 20 µl Lucifer yellow (Sigma Cat# L0259, 20 mg/ml stock solution in deionized water) in intracellular buffer before the recording for confirming the whole cell mode. Patch pipettes were visually guided using a MM-225 micromanipulator (Sutter Instrument, Navato, CA). Whole-cell recordings were made once a >5–10 GΩ seal was achieved. Cells were continuously superfused with HEPES buffer (125 mM NaCl, 25 mM HEPES, 10 mM D-glucose, 5 mM KCl, 2 mM CaCl2, 1 mM MgCl2; pH 7.2) and bath temperature was maintained at 32–33°C. Cells were first recorded at their resting membrane potential followed by recording at dialing membrane voltage to −65 mV holding potential for testing the action potential generations protocols.

### 2.8 Statistical analysis

Statistical tests were performed using GraphPad Prism software, Version 10.0.2. Data are presented as the means ± standard error of means (SEM). Differences between the 2 groups were examined using a unpaired t test (two-tailed), with p < 0.05 considered statistically significant. N = number of observations.

## 3 Results

### 3.1 LMMP identification, digestion, and neurosphere culture and differentiation

**Fig. 2:**
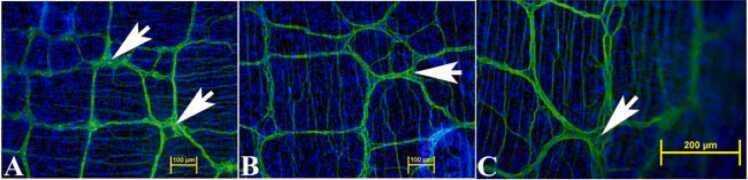
Immunofluorescence image of LMMP showing the extent of neural network in small intestine. The neural network was detected using neuron-specific anti-TUBB3 antibody (green) showing the distribution of neural processes running between the ganglia (arrows) from duodenum (A), jejunum (B) and ileum (C). The nuclei were stained with DAPI (blue). Scale bars, 100 µm (A, B) or 200 µm (C).

To maximize neurosphere yield, LMMP was isolated from entire small intestine by dissection in silicone dishes under stereo microscope. The presence of myenteric plexus in the LMMP samples from duodenum, jejunum and ileum were confirmed using neuron specific anti-TUBB3 antibody. Neural processes, cell bodies and ganglia were apparent throughout the small intestine (Fig. 2).

**Fig. 3:**
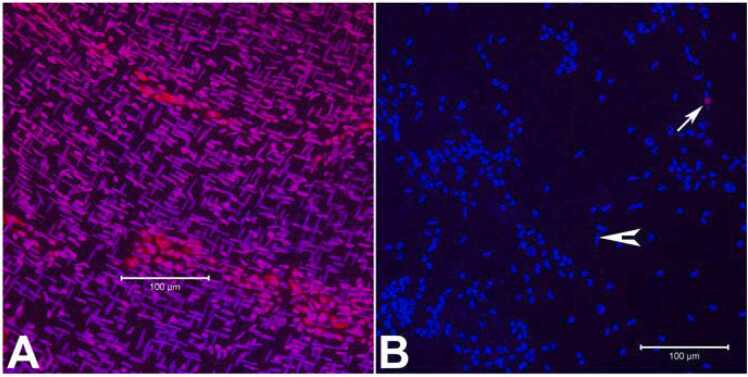
Live dead staining of LMMP samples using Propidium iodide. A: LMMP fixed, permeabilized and stained before 4 h digestion showing abundant long nuclei smooth muscle cells and some round nuclei cells; B: 4 h digested LMMP stained with Propidium iodide (red) showing mostly live round nuclei cells with occasional dead cells (arrow), cleared areas of muscle cells with few long nuclei smooth muscle cells (arrow head). A, B: merge of propidium iodide and DAPI (blue). Scale bar: 100 µm.

To determine the viability of the cells in the myenteric plexus after 4 h of enzymatic digestion, live-dead staining using propidium iodide was used. Patches of live myenteric plexus cells were apparent following the enzymatic digestion along with occasional long nuclei smooth muscle cells (arrow head) compared to the undigested LMMP (Fig. 3A, B). The LMMP after Liberase treatment revealed cleared areas of long nuclei smooth muscle cells and occasional presence of dead cells (≤0.3%, Fig. 3B, arrow).

**Fig. 4:**
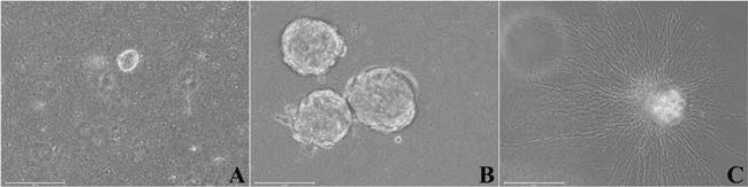
Phase contrast images of cultured enteric neurospheres from adult myenteric plexus in 3D matrigel-collagen matrix. A: Early neurosphere by day 3 of culture; B: Developed and undifferentiated neurosphere culture by day 7; C: Differentiated neurosphere sent out profuse neurites into the matrix and formed neurite mesh by day 10 of culture. Scale bars: 150 µm.

The dissociated adult myenteric plexus cells from one small intestine were suspended in the matrigel-collagen mix, cultured and differentiated in 3D embedded format in twelve 14 mm microwell of 35 mm Petri dishes. Small neurospheres (∼30 µm) formed within 2–3 days and becomes ∼150 µm sizes by 6–7 days in culture (Fig. 4 A, B) and remained undifferentiated. Total number of neurospheres per small intestine after 5–6 days in culture was 3284 (273.7 ± 46.5 per dish, mean ± SEM, n=6). In differentiation media, the neurospheres produce extensive neurites and formed neurite network (Fig. 4C) without any subculture.

### 3.2 Expression of neural stem and progenitor cell markers in early neurospheres

**Fig. 5:**
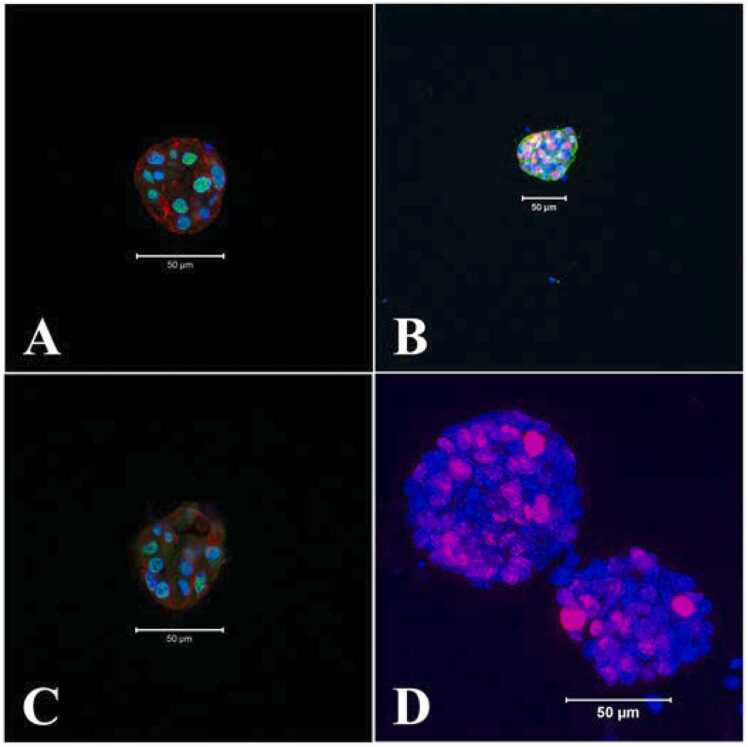
Expression of neural stem and progenitor cell markers in the growing neurospheres, day 4. A: Sox2, green, nuclear; B: Sox10, red, nuclear; C: Msi1, green, neuclear; D: Ki67, also a nuclear protein marker for proliferating cells, red, day 7. Counter stain DAPI (nucleus), blue and cytoplasmic alpha tubulin, red (A, C) or green (B). A, B, C: merge of blue, green and red. Scale bars: 50 µm. All neurospheres were positive to the labelling (Sox2, Sox10, Msi1, n = 3; Ki67, n = 4).

Sox2 and Sox10 are well characterized markers of neural stem and progenitor cells and are also required for their self-renewal (Amador-Arjona et al., 2015). (Kim et al., 2003, Pozniak et al., 2010). Both Sox2 and Sox10 were found to be highly expressed predominantly in the nuclei of the early neurospheres (Fig. 5A, B).

Additionally Msi1, the evolutionarily conserved pluripotent marker for neural stem and progenitor cells (Kanemura et al., 2001, Glazer et al., 2008) was also expressed in the growing neurospheres (Fig. 5C). These progenitor cells were distributed throughout the neurosphere body confirming the presence of these pluripotent cells in the early neurospheres. Active proliferation of neurosphere cells even at day 7 of growth was marked by presence of nuclear Ki67 expression (Fig. 5D).

**Fig. 6:**
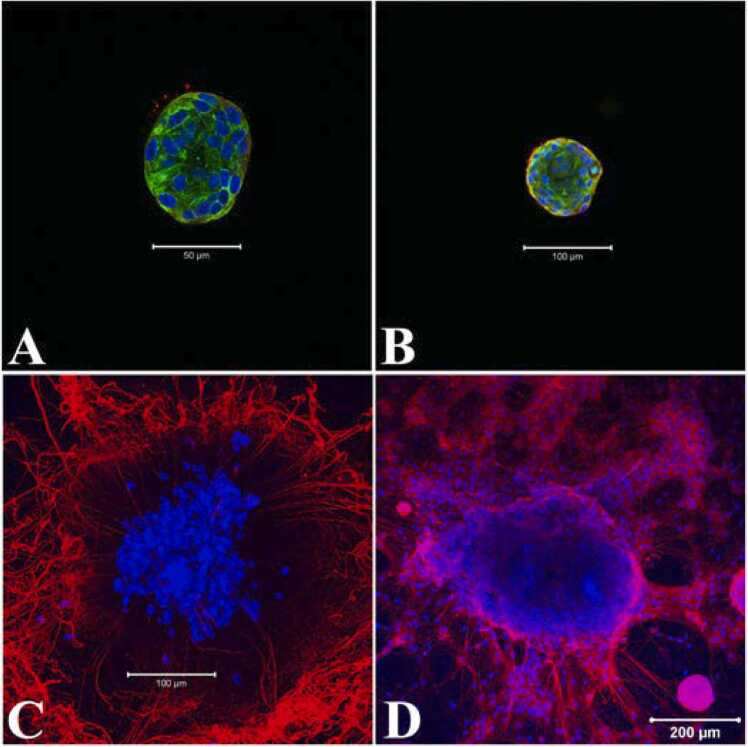
Expression of neural progenitor cell markers in early and differentiated neurospheres. A: Occasional expression of Nestin (red) in some peripheral cells, in the filamental area, day 4. B: Most of the peripheral cells showed expression of Nestin by day 5. C: Differentiated neurospheres with dispersed nuclei and nestin positive neurites, day 11. D: Neural progenitor cell marker p75^NTR^ (red) expressed in the dispersed neurons and in the neurospheres, at day 10 of growth media. Counter stain DAPI (nucleus), blue and cytoplasmic alpha tubulin, green. A, B, C: merge of blue, green and red. Scale bars: 50 µm (A), 100 µm (B, C) or 200 µm (D). All neurospheres were positive to the labelling (Nestin, n = 4; p75^NTR^, n = 5).

Nestin, an intermediate filament protein, is a marker of multipotent neural progenitor cells and is required for their self-renewal while persisting in mature neurons (Park et al., 2010; Hendrickson et al., 2011). In early undifferentiated neurospheres nestin expression was found to be restricted to the peripheral cells and occasionally in the fibers extending out from the periphery (Fig. 6A, B). Following 5 days in differentiation media the neurosphere showed dispersed nuclei with extensively outgrowing nestin positive neurites (Fig. 6C). The survival of the neuronal progenitors and their differentiation, migration and axonal outgrowth are also regulated the p75 neurotrophin receptor (p75^NTR^, Young et al., 2007; Meier et al., 2019). Examination of p75^NTR^ in late neurospheres confirmed its expression in the cell bodies, neurites and in the early neurons (Fig. 6D).

### 3.3 Neurosphere differentiation into neurons and neuronal subtypes

**Fig. 7:**
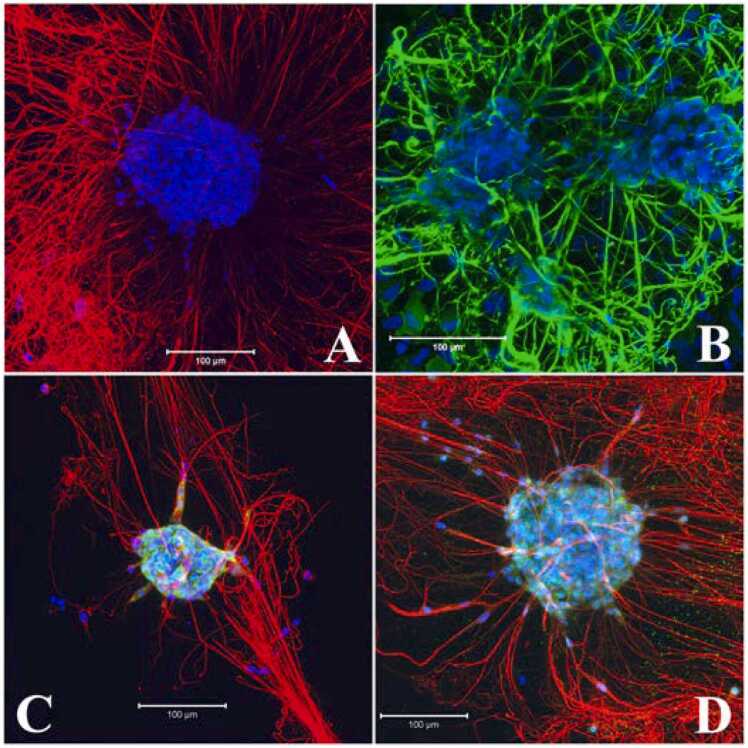
Expression of neuron specific proteins in differentiated neurospheres. A: 7 day differentiated neurosphere with extensive neuronal processes immunostained with Tubulin beta III (TUBB3) antibody (red). B: Abundant expression of the neuron specific protease PGP9.5 (green) in the neurites and cell bodies in 6 day differentiated neurosphere. C & D: Cell bodies of the peripheral cells of neurosphere were positive for RNA binding proteins, HuD/C and NeuN in 5 day differentiated neurospheres (green). Counterstain, DAPI (nucleus), blue and alpha tubulin (C, D) red. C, D: merge of blue, green and red. Scale bars: 100 µm. All neurospheres were positive to the labelling (TUBB3, PGP9.5, n = 3; HuD/C, NeuN, n = 2).

After 5–7 days in growth media, neurospheres developed in the matrigel-collagen matrix were grown in differentiation media for 5–8 days. Neurites were observed within 2–3 days. The differentiation of neurons in the neurospheres was confirmed by expression of neuron-specific protein Tubulin beta III specifically along the neurites (Fig. 7A or 29 days in vitro, data not shown) and the pan-neuronal marker PGP9.5 in the cell bodies and neuronal processes (Fig. 7B). The neuronal processes formed mesh-like structures around the differentiated neurospheres. Another pan-neural RNA binding protein HuD/C that does not localize to the neural processes (Desmet et al., 2014) showed cytoplasmic and nuclear localization in the differentiated neurospheres (Fig. 7C). NeuN, a neuronal specific RNA binding protein (Mullen, 1992) also showed nuclear and cytoplasmic expression following 5 days in differentiation media (Fig. 7D). Many of the HuD/C and NeuN positive cells in the differentiated neurospheres generated neurites detected by alpha Tubulin antibody (Fig. 7C, D) which colocalized with neuron-specific Tubulin beta III positive neurites (data not shown).

**Fig. 8:**
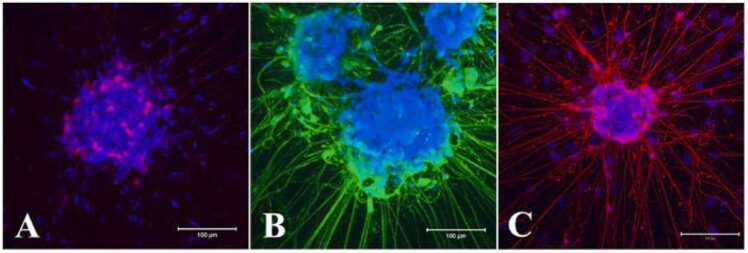
Neurospheres differentiate into neural subtypes following 8 days in differentiation media. A: Calcium binding calretinin expressed primarily in the cell bodies (red). B & C: Both ChAT (B, green) and nNOS (C, red) showed expression in cell bodies and neurites. Nuclei are stained with DAPI in blue. Scale bars: 100 µm. IgG control antibodies produced no signals (data not shown). All neurospheres were positive to the labelling (Calretinin, nNOS, n = 3; ChAT, n = 4).

In addition to the differentiation of neurons in the neurosphere, the Ca^2+^-binding protein Calretinin, known to be predominantly expressed in specific neurons of the central and peripheral nervous system (Schwaller, 2014), was also detected in the cell bodies in differentiated neurospheres (Fig. 8A). Putative cholinergic neuronal marker choline acetyltransferase (ChAT) that produces acetylcholine (Granger et al., 2020) was expressed both in the neurites and cell bodies of neurospheres differentiated for 8 days (Fig. 8B). Neuronal nitric oxide synthase (nNOS), which generates nitric oxide (Echagarruga et al., 2020), was expressed both in the cell bodies and processes of neurons in differentiated neurospheres (Fig. 8C). The isotype control antibodies produced no signal (data not shown).

### 3.4 Whole-cell patch clamp determination of action potentials in differentiated neurospheres and neurons

**Fig. 9:**
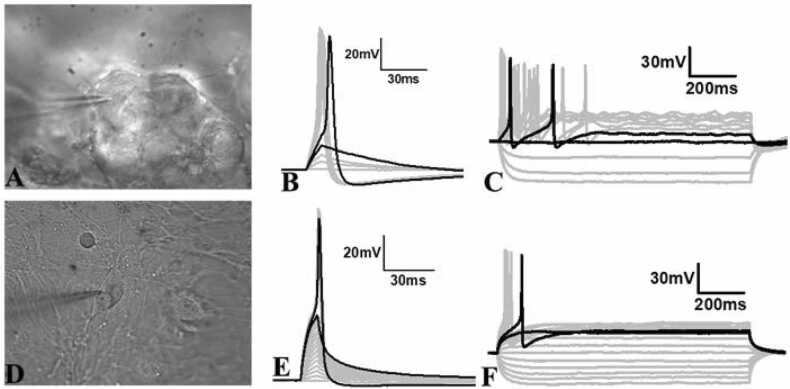
Light micrograph of a single cell in a neurosphere (A) or a differentiated neuron (D) with a patch pipette recording the action potential firing ability of a single cell. B & E: Representative traces of membrane voltage in response to the short depolarization current pulses (B: 1–100 pA or E: 1–200 pA for 2 ms with 10 pA increment each time) from a patched cell within a neurosphere (A) or a differentiated neuron (D). Black traces represent membrane responses to two consecutive stimuli before (shorter peak) and after (larger peak) firing the first action potential. Gray traces below the short peak black trace show the passive response of the membrane to subthreshold stimuli. Gray traces with a large peak represent the fringe of action potential each time with a higher magnitude current than the threshold current. C & F: Representative traces of membrane voltage in response to longer hyper and depolarization current steps (C: Steps −40 – 70pA, F: −50 – 70 pA) with 10pA increment in subsequent steps for 1100 ms from a patched cell in A or D. Black traces represent membrane responses to two consecutive stimuli before (shorter peak) and after (larger peak) firing the first action potential.

We performed whole-cell patch-clamp recordings to investigate the ability of the neurons in the neurospheres to generate action potentials (Fig. 9A), as well as those that migrate from the neurosphere (Fig. 9D) after 1–2 weeks in differentiation media. Notably, the presence of matrigel-collagen matrix affected our ability to readily approach the cell surface and form a gigaohm seal on the cell membrane, but in the successful attempts, we made a gigaohm seal (1–5 GΩ) and subsequently recorded neuronal activity. Cells present in the neurosphere (Fig. 9A) or away from the neurosphere (Fig. 9D) showed a wide range of resting membrane potential (-30 to −55 mV) similar to the typical neurons as reported in the literature (Bean, 2007, Tripathy et al., 2014), and showed no spontaneous firing of action potentials. To assess whether these cells are capable of generating action potentials, we employed brief pulses of depolarizing currents and recorded membrane potential. In both instances, neurons inside and outside of the neurosphere showed a linear passive increase in the membrane potential (Fig. 9B, E, small gray traces) until the membrane reached the firing threshold (Fig. 9B, E, black traces showing before and after threshold) and generated action potentials with each subsequent increment in the magnitude of injected current pulse (Fig. 9B, E, large gray traces). In addition, we recorded the membrane voltage in response to multiple long-step currents of hyper- and de-polarization polarity to confirm action potential generation. Long-step current injections reliably generated a few action potentials in a few neurons (Fig. 9C) and only a single in others (Fig. 9F) suggesting that neurons are able to generate action potentials.

**Fig. 10:**
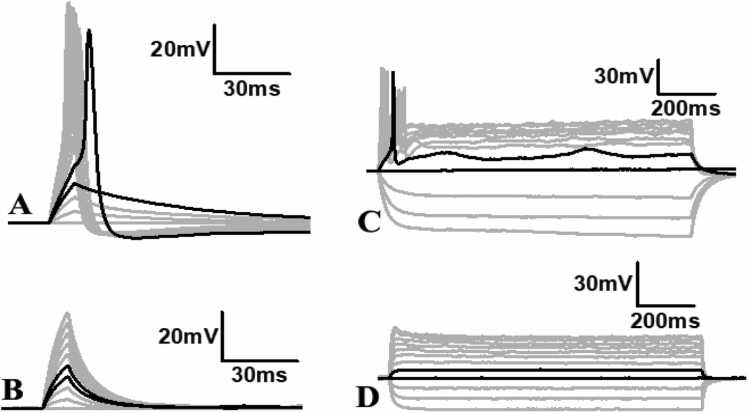
Representative traces of membrane voltage in response to the short pulse stimulation (A, B; 1–100 pA for 2 ms with 10 pA increment) or long step stimulation (C, D; −40 – 70 pA with 10 pA increment for 1100 ms) depolarization currents. Black traces represent membrane responses to two consecutive stimuli before (shorter peak) and after (larger peak) firing the first action potential. Gray traces below short peak black trace show passive response of the membrane to subthreshold stimuli. Gray traces with a large peak represent the fringe of action potential each time with a higher magnitude current than the threshold current. B, D: Representative traces of membrane voltage after incubation of cells in 2 µm Tetradotoxin for 5 min. The two black traces represent the two consecutive stimuli responses as seen in B or D, however without any action potential generation. None of the higher magnitude currents than the threshold current showed any action potential.

Finally, to confirm whether the action potential generated upon a depolarization pulse or prolonged step current is mediated by voltage-gated sodium channels, we applied 2 µm Tetrodotoxin (TTX) in the bath after recording action potential from a neuron (Fig. 10A, C). Within 5 min of TTX addition, the action potential generation was completely abolished (Fig. 10B, D). Notably the same neurons showed action potential before TTX addition (Fig. 10A, C), suggesting that the action potential is mediated by voltage-gated sodium channels identical to that of a typical neuron. These data suggest that neurons within and outside of the neurosphere are able to fire action potentials and these are mediated by voltage-gated sodium channels.

### 3.5 Non-neuronal cell types in neurospheres

**Fig. 11:**
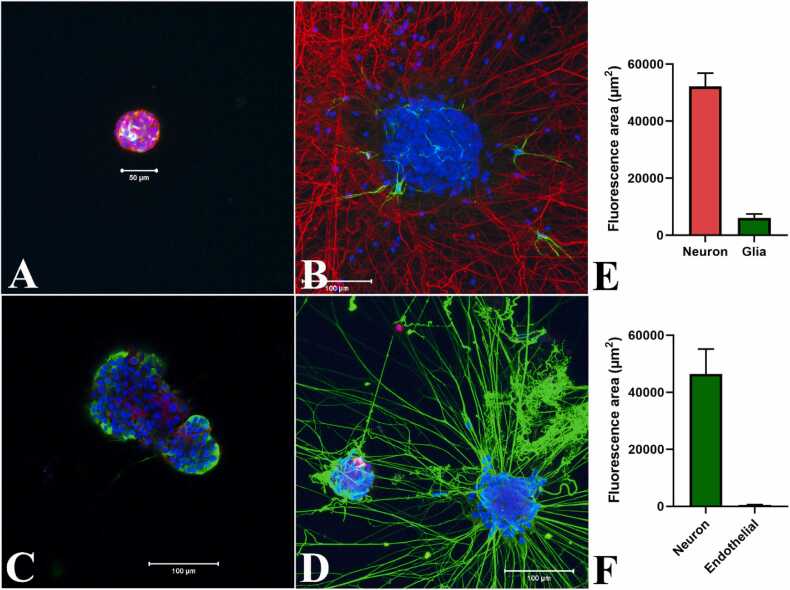
Immunofluorescence showing formation of neuronal and non-neuronal cells in growing and differentiated neurospheres. Undifferentiated neurospheres were fixed either in 4 (A) or 6 (C) days in culture and differentiated neurospheres were probed following 7 (B) or 3 (D) days in culture. A: Occasional expression of GFAP (yellow) in early neurospheres; cytoplasmic stain, alpha tubulin (red). B: Development of GFAP positive glial cells (green) in the TUBB3 positive neuronal processes (red) of differentiated neurosphere. C: Expression of VE-cadherin (red) mainly in the non-peripheral cells of undifferentiated neurosphere while TUBB3 (green) producing neurons without neurites were mostly peripheral. D: Formation of VE-cadherin positive vascular endothelial cells in some differentiated neurosphere connected with TUBB3 positive neurites. Neuclear stain: DAPI, blue. All images are merge of blue, green and red. Scale bars: 50 µm (A) or 100 µm (B, C, D). The relative fluorescence area measurement of the differentiated neurosphere images using hybrid cell count software, analyzed by the BZ-H4C application (Keyence), revealed 10.4% glia (E, neuron 89.6%, P < 0.001, n =3) and 1.3% endothelial cell (F, neuron 98.7%, P < 0.035, n = 2) expression over the neurons with TUBB3 stained neurites. Data represent the means ± standard error of means. n = number of observations.

Glial cells are central components of neurogenic niches in the embryonic and adult central nervous system (Falk and Götz, 2017). Glial cells also orchestrate many important aspects of nervous system formation and function (Allen and Lyons, 2018). Examination of early undifferentiated neurospheres showed sporadic expression of GFAP, a glia-specific protein (Fig. 11A). Differentiated neurospheres with extensive neurite networks revealed only a limited occurrence of GFAP positive glial cells (Fig. 11B) following 7 days in differentiation media. The Glia derived GFAP mean fluorescence area was 6080 ± 1402 µm^2^ compared to 52132 ± 4765 µm^2^ for TUBB3 positive neurons in the differentiated neurospheres representing about 10.4% of the total neuron-glia fluorescence area (P < 0.001, n = 3).

Endothelial cell released factors are known to enhance neural progenitor cell proliferation and differentiation (Shen et al., 2004, Gama Sosa et al., 2007). Vascular endothelial cells also promote neurite outgrowth, enhanced synapse function and accelerated electrophysiological development of neurons (Wu et al., 2017; Grasman et al., 2017). Vascular endothelial cadherin expression confirmed the development of endothelial cells mainly in the core of neurospheres before differentiation while immature neurons expressing TUBB3 were mostly peripheral (Fig. 11C). Only few endothelial cells were detected in the neurospheres following 3 days in differentiation media(Fig. 11D). The vascular endothelia derived cadherin mean fluorescence area was 593.5 ± 13.5 µm^2^ compared to 46,413 ± 8785 µm^2^ for the TUBB3 positive differentiated neurons representing about 1.3 % of the total fluorescence area (P < 0.035, n = 2).

## 4 Discussion

The objective of this study was the development of a novel murine culture system for characterization of enteric neurons, neurochemically defined sybtypes and their functional properties using immunofluorescence assays and electrophysiological studies following differentiation of adult myenteric neural stem/progenitor cells. Here, we describe the development of an enteric neurosphere culture in 3D format from the ganglionated myenteric plexus of adult mice that differentiates predominantly into functionally active neuronal subtypes along with limited glial and endothelial cells. The isolation of enteric nervous system cells can be challenging as the myenteric plexus is located between the longitudinal and circular smooth muscle cells. To initiate the neurosphere culture, ganglionated longitudinal muscle myenteric plexus was isolated from the small intestine of adult mice (Fig. 1) under DPBS to prevent drying of the LMMP samples during the isolation process. The average time to isolate the entire LMMP from one longitudinally open small intestine was about 20–25 min and resulted in no bacterial growth in the cultures. The starting LMMP samples were initially validated to have ganglionated myenteric plexus from duodenum, jejunum and ileum using neuron specific TUBB3 immunofluorescence analyses (Fig. 2). Usually, LMMP samples are digested first by collagenase followed by trypsin (Smith et al., 2013; Wahba et al., 2016; Zhang et al., 2021). In this study, to remove the longitudinal muscle cells from myenteric plexus, the LMMP pieces were digested in a one-step process using a cocktail of Collagenase I, Collagenase II, a neutral protease, Thermolysin (Liberase TH) and DNase I for 4 h to improve the abundance of intrinsic neural stem cells (Grundmann et al., 2015) as the myenteric ganglia do not utilize collagen but glia cells for tissue adhesion (Gershon et al., 1991). The prolonged Liberase TH digestion of LMMP samples produced only occasional dead cells (Fig. 3) along with cleared areas of muscle cells, as observed by Grundmann et al., (2015) using scanning electron microscopy and western blot analysis. Enteric neurons are terminally differentiated cells. Culture of primary neurons usually results in relatively lower yields of enteric neurons (Smith et al., 2013; Wahba et al., 2016; Brun et al., 2018), however culture and differentiation of enteric neural stem/progenitor cells produces large number of enteric neurons (Zhang et al., 2021). Usually enteric neural stem/progenitor cells are cultured in matrix uncoated plates for neurosphere growth and then separately cultured for neurosphere differentiation to produce neurons in matrix coated format (Almond et al., 2007; Binder et al., 2015; Grundmann et al., 2015; Hotta et al., 2016; Zhang et al., 2021). To improve the yield of neurospheres, we cultured the enteric neural stem/progenitor cells in 3D matrix for both growth and differentiation steps in single dish containing matrigel and collagen matrix which has shown to improve cell attachment (Wahba et al., 2016). The dissociated myenteric plexus cells from one small intestine was enough for twelve 14 mm microwell of 35 mm MatTek dish cultures and improved the total neurosphere count by 1.6–2.2 fold compared to the standard matrix non-coated culture methods (Zhang et al., 2013; Zhang et al., 2021). The higher yield of neurospheres could be attributed due to the single step Liberase TH digestion which produces hundreds of myenteric networks (Grundmann et al., 2015) while avoiding excessive cell death (Fig. 3), elimination of possible loss of stem/progenitor cells from the 3D matrix during media change, and addition of GDNF in the growth media because of its essential role in the early survival and proliferation of enteric neural crest cells in the small bowel and colon (Gianino et al., 2003; Hao et al., 2009).

Within the Matrigel, formation of neurospheres were apparent within ∼3 days of culture and remained undifferentiated until induced for differentiation by 6–7 days (Fig. 4). Analysis of the cellular nature of early undifferentiated neurospheres confirmed the expression of well-established neural stem and progenitor cell markers, Sox2 and Msi1 throughout the neurosphere (Fig. 5). Beyond self-renewal properties, Sox2 also plays a role in proper activation of neural differentiation (Amador-Arjona et al., 2015) while Msi1 modulates progenitor cell expansion (Kanemura et al., 2001, Wang et al., 2008). Also, the expression of Sox10 in early neurospheres underscored the potential of glia cell formation during neurosphere differentiation (Fig. 11) as this transcription factor is the key regulator of glial cell development (Britsch et al., 2001, Liu et al., 2020). On the contrary Nestin, a cytoskeletal intermediate filament protein of neural progenitor cells (Park et al., 2010; Bernal et al., 2018) was barely expressed in day 4 neurospheres but found to be highly distributed in the neurites of differentiated neurons (Fig. 6A-C) as observed in mature neurons of adult rat and human brain (Hendrickson et al., 2011). To overcome apoptosis-mediated neuronal losses in myenteric ganglia, new neurons are formed from the dividing precursor cells which express both nestin and p75^NTR^ (Kulkarni et al., 2017). Similar to nestin expression profile in growing neurospheres, both small and large neurospheres were found to be neurogenic (Young et al., 2007) as confirmed by expression of p75^NTR^ (Fig. 6D).

Neuronal differentiation of neurospheres was evident from expression of neuron specific markers Tubulin beta III, PGP9.5, HuD/C and NeuN proteins within 3–7 days of culture in differentiation media (Fig. 7). In LMMP dissociated 2D cultures, neuronal morphology and immunocytochemical features also became apparent after 7–10 days in culture (Smith et al., 2013; Wahba et al., 2016). Some of the major myenteric neural subtypes e.g., ChAT, Calretinin and nNOS first arrive in the myenteric plexus by embryonic day E13.5 or earlier (Bergner et al., 2014, Erickson et al., 2014). Interestingly, all these myenteric neuron subtypes appeared in the differentiated neurosphere following 8 days in culture (Fig. 8) as observed in dissociated neurosphere subtype differentiation in vitro (Binder et al., 2015, Hotta et al., 2016, Cheng et al., 2017). In matrix coated dissociated neuron culture format, expression of calretinin was reported after about 10 days in culture (Smith et al., 2013) when ChAT was observed around 21 days in vitro (Wahba et al., 2016). Enteric neurospheres are usually grown in matrix coated or non-coated dishes containing EGF and bFGF, and for cell differentiation on matrix coated plates in absence of growth factors (Almond et al., 2007; Metzger et al., 2009; Binder et al., 2015; Grundmann et al., 2015; Hotta et al., 2016; Zhang et al., 2021). The present study demonstrated that the myenteric neural stem/progenitor cells can be cultured to form neurospheres in matrix embedded format and can also be differentiated to form neurons and myenteric neural subtypes in the same dish (Figs. 7 and 8). This method also incorporated GDNF in both the growth and differentiation media for neurogenesis and differentiation because this factor promotes the proliferation, migration and neural differentiation of enteric neural crest cells (Almond et al., 2007; Hao et al., 2009; Uesaka et al., 2013; Cheng et al., 2016; McKeown et al., 2017).

The differentiated neurospheres and neurons showed the defining feature of firing action potentials and revealed a wide range of resting membrane potential (-30 to −55mv), suggesting a heterogeneous population likely to be at different stages of maturity (Ramoa et al., 1994). At their resting membrane potential none of the cells showed any spontaneous action potential which is very common in *in vitro* systems due to the absence of neuronal network connectivity. However, when we stimulated neurons using a current-clamp approach, we observed passive changes in membrane potential with subthreshold stimuli and consistent action potential firing upon reaching the threshold stimulation and subsequently higher stimuli. The membrane voltage responses following stimulation by longer hyper and depolarization currents also generated few or single action potential spike patterns which suggest a heterogeneous population likely to comprise different subtypes of neurons as seen in our immunocytochemistry results. Similar to the action potentials of the CNS neurons which are mediated by tetrodotoxin-sensitive voltage-gated sodium channels, we observed a complete blockade of action potential in the patched neurons within 5 min of tetrodotoxin application (Figs. 9 and 10), confirming their physiological similarities with CNS neurons.

In addition to neurons, differentiated neurospheres contained differentiated glial cells representing only ∼10% of the total neuron-glia specific protein fluorescence (Fig. 11). The enteric neural stem cells derived from neural crest cells (Dupin et al., 2012) are present in the neurospheres and differentiate into neuron and glia cells *in vivo* (Hotta et al., 2013; Burns et al., 2014). Both FACS selected or unselected neural crest derived cells form neurospheres with abundant glia cells before and after differentiation (Binder et al., 2015, Cheng et al., 2017). Like peripheral glia cells, Sox10 is required for the development of enteric glia cells (Britsch et al., 2001, Paratore et al., 2001). Even in the presence of a non-limiting Sox10 expression in early neurospheres, the limited glia cell expression could be attributed to the presence of GDNF during both proliferation and differentiation of neurospheres as GDNF availability determines enteric neuron numbers by controlling ENS precursor proliferation (Gianino et al., 2003; McKeown et al., 2017; Cheng et al., 2016). In addition to glia cells, the presence of endothelial cells in the neurospheres was found to be very interesting as endothelial cells promote neural stem cell proliferation, differentiation and functional maturation of neuron through activation of VEGF signaling (Sun et al., 2010; Wu et al., 2017). It would be intriguing through future clonal analysis to determine whether the endothelial cells differentiate from a common multipotent stem cell or from endothelial progenitor cells (Jing et al., 2022). The use of ENS culture in mice models to study the function of ENS in health and disease has been instrumental to advance our present understanding of ENS physiology and disease development (Schonkeren et al., 2022). Now with the advent of this functional neurosphere culture, intestinal organoids or enteric mesenchymal cells can be cocultured to study cell-cell molecular interaction pathways which can be difficult to analyze in vivo. The availability to manipulate the developmental components of this functional ENS culture system presents an advantage to study the ENS in health and disease.

## 5 Conclusion

The method we describe involves the isolation of LMMP from flattened small intestine and its digestion and growth in conditions that enhance neurosphere production. This neurosphere culture method is a single step procedure for growing adult myenteric ganglia in a 3D format for neural growth and differentiation. The developed neurospheres produce myenteric neuronal subtypes and functionally active neurons within 8 days of differentiation and can be cultured for at least 4 weeks. The differentiated neurospheres also contain a limited number of enteric glia and endothelial cells.

## Funding

C.M. was supported by 10.13039/100000002NIH Research Training Program (NIH 5T32HL007849–22). D.E.L. was supported by the iTHRIV Scholars Program which is funded in part by the Natioal Center of Advancing Translational Sciences of the National Institute of Health under award numbers UL1TR003015 and KL2TR003016. D.E.L. was also supported by the a grant from the Departmet of Surgery, University of Virginia. S.R/ M. was supported by a grant from Bill & Melinda Gates Foundation investment grant ID# INV-039470.

## CRediT authorship contribution statement

**Sean R. Moore:** Conceptualization, Formal analysis, Funding acquisition, Investigation, Project administration, Resources, Supervision, Validation, Visualization, Writing – review & editing. **Daniel Levin:** Conceptualization, Formal analysis, Funding acquisition, Investigation, Methodology, Project administration, Resources, Supervision, Validation, Visualization, Writing – review & editing. **Bhanu P. Tewari:** Writing – review & editing, Writing – original draft, Visualization, Validation, Supervision, Resources, Methodology, Investigation, Formal analysis, Data curation. **Allan M. Goldstein:** Visualization, Validation, Supervision, Investigation, Formal analysis, Writing – review & editing. **Harald Sontheimer:** Data curation, Formal analysis, Investigation, Methodology, Project administration, Resources, Supervision, Validation, Visualization, Writing – review & editing. **Lily Cheng:** Formal analysis, Investigation, Methodology, Project administration, Resources, Validation, Visualization, Writing – review & editing. **Arabinda Mandal:** Writing – review & editing, Writing – original draft, Conceptualization, Data curation, Formal analysis, Investigation, Methodology, Supervision, Validation, Visualization. **Chioma Moneme:** Writing – original draft, Visualization, Methodology, Investigation, Formal analysis, Data curation.

## Declaration of Competing Interest

The authors declare that they have no known competing financial interests or personal relationships that could have appeared to influence the work reported in this paper.

## Data Availability

Data will be made available on request.

## References

[bib1] Allen N.J., Lyons D.A. (2018). Glia as architects of central nervous system formation and function. Science.

[bib2] Almond S., Lindley R.M., Kenny S.E., Connell M.G., Edgar D.H. (2007). Characterisation and transplantation of enteric nervous system progenitor cells. Gut.

[bib3] Amador-Arjona Alejandro, Cimadamore Flavio, Huang Chun-Teng, Wright Rebecca, Lewis Susan, Gage Fred H., Terskikh Alexey V. (2015). SOX2 primes the epigenetic landscape in neural precursors enabling proper gene activation during hippocampal neurogenesis. Proc. Natl. Acad. Sci. USA.

[bib4] Aubé A.-C., Cabarrocas J., Bauer J., Philippe D., Aubert P., Doulay F., Liblau R., Galmiche J.P., Neunlist M. (2006). Changes in enteric neurone phenotype and intestinal functions in a transgenic mouse model of enteric glia disruption. Gut.

[bib5] Bagyánszki M.ária, Bódi Nikolett (2012). Diabetes-related alterations in the enteric nervous system and its microenvironment. World J. Diabetes.

[bib6] Bean Bruce P. (2007). The action potential in mammalian central neurons. Nat. Rev. Neurosci..

[bib7] Bergner Annette J., Stamp Lincon A., Gonsalvez David G., Allison Margaret B., Olson David P., Myers Jr Martin G., Anderson Colin R., Young Heather M. (2014). Birthdating of myenteric neuron subtypes in the small intestine of the mouse. J. Comp. Neurol..

[bib8] Bernal Aurora, Arranz Lorena (2018). Nestin-expressing progenitor cells: function, identity and therapeutic implications. Cell Mol. Life Sci..

[bib9] Binder Ellen, Natarajan Dipa, Cooper Julie, Kronfli Rania, Cananzi Mara, Delalande Jean-Marie, McCann Conor, Burns Alan J., Thapar Nikhil (2015). Enteric neurospheres are not specific to neural crest cultures: implications for neural stem cell therapies. PLoS One.

[bib10] Bondurand N., Natarajan D., Thapar N., Atkins C., Pachnis V. (2003). Neuron and glia generating progenitors of the mammalian enteric nervous system isolated from foetal and postnatal gut cultures. Development.

[bib11] Britsch S., Goerich D.E., Riethmacher D., Peirano R.I., Rossner M., Nave K.A., Birchmeier C., Wegner M. (2001). The transcription factor Sox10 is a key regulator of peripheral glial development. Genes Dev..

[bib12] Brun Paola, Akbarali Hamid I. (2018). Culture of neurons and smooth muscle cells from the myenteric plexus of adult mice. Methods Mol. Biol..

[bib13] Burns Alan J., Thapar Nikhil (2014). Neural stem cell therapies for enteric nervous system disorders. Nat. Rev. Gastroenterol. Hepatol..

[bib14] Bush T.G., Savidge T.C., Freeman T.C., Cox H.J., Campbell E.A., Mucke L., Johnson M.H., Sofroniew M.V. (1998). Fulminant jejuno-ileitis following ablation of enteric glia in adult transgenic mice. Cell.

[bib15] Cheng Lily S., Graham Hannah K., Pan Wei Hua, Nagy Nandor, Carreon-Rodriguez Alfonso, Goldstein Allan M., Hotta Ryo (2016). Optimizing neurogenic potential of enteric neurospheres for treatment of neurointestinal diseases. J. Surg. Res.

[bib16] Cheng Lily S., Hotta Ryo, Graham Hannah K., Belkind-Gerson Jaime, Nagy Nandor, Goldstein Allan M. (2017). Postnatal human enteric neuronal progenitors can migrate, differentiate, and proliferate in embryonic and postnatal aganglionic gut environments. Pedia Res..

[bib17] Cornet A., Savidge T.C., Cabarrocas J., Deng W.L., Colombel J.F., Lassmann H., Desreumaux P., Liblau R.S. (2001). Enterocolitis induced by autoimmune targeting of enteric glial cells: a possible mechanism in Crohn's disease?. Proc. Natl. Acad. Sci. USA.

[bib18] Desmet A.-S., Cirillo C., Vanden Berghe P. (2014). Distinct subcellular localization of the neuronal marker HuC/D reveals hypoxia-induced damage in enteric neurons. Neurogastroenterol. Motil..

[bib19] Dupin Elisabeth, Sommer Lukas (2012). Neural crest progenitors and stem cells: from early development to adulthood. Dev. Biol..

[bib20] Echagarruga Christina T., Gheres Kyle W., Norwood Jordan N., Drew Patrick J. (2020). nNOS - expressing interneurons control basal and behaviorally evoked arterial dilation in somatosensory cortex of mice.. Elife.

[bib21] Erickson C.S., Lee S.J., Barlow-Anacker A.J., Druckenbrod N.R., Epstein M.L., Gosain A. (2014). Appearance of cholinergic myenteric neurons during enteric nervous system development: comparison of different ChAT fluorescent mouse reporter lines. Neurogastroenterol. Motil..

[bib22] Falk S., Götz M. (2017). Glial control of neurogenesis. Curr. Opin. Neurobiol..

[bib23] Fleming II Mark A., Ehsan Lubaina, Moore Sean R., Levin Daniel E. (2020). The Enteric Nervous System and Its Emerging Role as a Therapeutic Target. Gastroenterol. Res Pr..

[bib24] Furness John B. (2012 Mar 6). The enteric nervous system and neurogastroenterology. Nat. Rev. Gastroenterol. Hepatol..

[bib25] Gama Sosa Miguel A., De Gasperi Rita, Rocher Anne B., Perez Gissel M., Simons Keila, Cruz Daniel E., Hof Patrick R., Elder Gregory A. (2007). Interactions of primary neuroepithelial progenitor and brain endothelial cells: distinct effect on neural progenitor maintenance and differentiation by soluble factors and direct contact. Cell Res.

[bib26] Gershon M.D., Rothman T.P. (1991). Enteric glia. Glia.

[bib27] Gianino Scott, Grider John R., Cresswell Jennifer, Enomoto Hideki, Heuckeroth Robert O. (2003). GDNF availability determines enteric neuron number by controlling precursor proliferation. Development.

[bib28] Glazer Robert I., Wang Xiao-Yang, Yuan Hongyan, Yin Yuzhi (2008). Musashi1: a stem cell marker no longer in search of a function. Cell Cycle.

[bib29] Granger Adam J., Wang Wengang, Robertson Keiramarie, El-Rifai Mahmoud, Zanello Andrea F., Bistrong Karina, Saunders Arpiar, Chow Brian W., Nuñez Vicente, Turrero García Miguel, Harwell Corey C., Gu Chenghua, Sabatini Bernardo L. (2020). Cortical ChAT^+^ neurons co-transmit acetylcholine and GABA in a target- and brain-region-specific manner. Elife.

[bib30] Grasman J.M., Kaplan D.L. (2017). Human endothelial cells secrete neurotropic factors to direct axonal growth of peripheral nerves. Sci. Rep..

[bib31] Grundmann David, Klotz Markus, Rabe Holger, Glanemann Matthias, Schäfer Karl-Herbert (2015). Isolation of high-purity myenteric plexus from adult human and mouse gastrointestinal tract. Sci. Rep..

[bib32] Gulbransen B.D., Sharkey K.A. (2012). Novel functional roles for enteric glia in the gastrointestinal tract. Nat. Rev. Gastroenterol. Hepatol..

[bib33] Hao Marlene M., Young Heather M. (2009). Development of enteric neuron diversity. J. Cell Mol. Med.

[bib34] Hendrickson Michael L., Rao Abigail J., Demerdash Omar N.A., Kalil Ronald E. (2011). Expression of nestin by neural cells in the adult rat and human brain. PLoS One.

[bib35] Hotta R., Cheng L.S., Graham H.K., Pan W., Nagy N., Belkind-Gerson J., Goldstein A.M. (2016). Isogenic enteric neural progenitor cells can replace missing neurons and glia in mice with Hirschsprung disease. Neurogastroenterol. Motil..

[bib36] Hotta Ryo, Stamp Lincon A., Foong Jaime P.P., McConnell Sophie N., Bergner Annette J., Anderson Richard B., Enomoto Hideki, Newgreen Donald F., Obermayr Florian, Furness John B., Young Heather M. (2013). Transplanted progenitors generate functional enteric neurons in the postnatal colon. J. Clin. Invest.

[bib37] Jing Jingti, Jiang Haoming, Zhang Lin (2022). Endothelial progenitor cells promote neural stem cell proliferation in hypoxic conditions through VEGF via the PI3K/AKT pathway. J. Recept Signal Transduct. Res..

[bib38] Kanemura Y., Mori K., Sakakibara S., Fujikawa H., Hayashi H., Nakano A., Matsumoto T., Tamura K., Imai T., Ohnishi T., Fushiki S., Nakamura Y., Yamasaki M., Okano H., Arita N. (2001). Musashi1, an evolutionarily conserved neural RNA-binding protein, is a versatile marker of human glioma cells in determining their cellular origin, malignancy, and proliferative activity. Differentiation.

[bib39] Kim Jaesang, Lo Liching, Dormand Emma, Anderson David J. (2003). SOX10 maintains multipotency and inhibits neuronal differentiation of neural crest stem cells. Neuron.

[bib40] Kruger Genevieve M., Mosher Jack T., Bixby Suzanne, Joseph Nancy, Iwashita Toshihide, Morrison Sean J. (2002). Neural crest stem cells persist in the adult gut but undergo changes in self-renewal, neuronal subtype potential, and factor responsiveness. Neuron.

[bib41] Kulkarni S., Micci M.-A., Leser J., Shin C., Tang S.-C., Fu Y.-Y., Liu L., Li Q., Saha M., Li C., Enikolopov G., Becker L., Rakhilin N., Anderson M., Shen X., Dong X., Butte M.J., Song H., Michelle Southard-smith E. (2017). Michelle Southard-Smith, Raj P Kapur, Milena Bogunovic, Pankaj J Pasricha. Adult enteric nervous system in health is maintained by a dynamic balance between neuronal apoptosis and neurogenesis. Proc. Natl. Acad. Sci. USA.

[bib42] Kun-Wei Wu Jia-Lin Mo, Zeng-Wei Kou Qi. Liu, Ling-Ling Lv Yu. Lei, Feng-Yan Sun Neurovascular (2017). Interaction Promotes the Morphological and Functional Maturation of Cortical Neurons. Front Cell Neurosci..

[bib43] Levin Daniel E., Mandal Arabinda, Fleming Mark A., Bae Katherine H., Gerry Brielle, Moore Sean R. (2020). Intestinal crypt-derived enteroid coculture in presence of peristaltic longitudinal muscle myenteric plexus. Biol. Methods Protoc..

[bib44] Liddle Rodger A. (2018). Parkinson's disease from the gut. Brain Res..

[bib45] Liu Jessica Aijia, Tai Andrew, Hong Jialin, Cheung May Pui Lai, Sham Mai Har, Cheah Kathryn S.E., Cheung Chi Wai, Cheung Martin (2020). Fbxo9 functions downstream of Sox10 to determine neuron-glial fate choice in the dorsal root ganglia through Neurog2 destabilization. Proc. Natl. Acad. Sci. USA.

[bib46] Lomax A.E., Fernández E., Sharkey K.A. (2005 Feb). Plasticity of the enteric nervous system during intestinal inflammation. Neurogastroenterol. Motil..

[bib47] Malik Astha, Kondratov Roman V., Jamasbi Roudabeh J., Geusz Michael E. (2015). Circadian clock genes are essential for normal adult neurogenesis, differentiation, and fate determination. PLoS One.

[bib48] McKeown Sonja J., Mohsenipour Mitra, Bergner Annette J., Young Heather M., Stamp Lincon A. (2017). Exposure to GDNF enhances the ability of enteric neural progenitors to generate an enteric nervous system. Stem Cell Rep..

[bib49] McQuade Rachel M., Singleton Lewis M., Wu Hongyi, Lee Sophie, Constable Remy, Di Natale Madeleine, Ringuet Mitchell T., Berger Joel P., Kauhausen Jessica, Parish Clare L., Finkelstein David I., Furness John B., Diwakarla Shanti (2021). The association of enteric neuropathy with gut phenotypes in acute and progressive models of Parkinson's disease. Sci. Rep..

[bib50] Meier Sonja, Alfonsi Fabienne, Kurniawan Nyoman D., Milne Michael R., Kasherman Maria A., Delogu Alessio, Piper Michael, Coulson Elizabeth J. (2019). The p75 neurotrophin receptor is required for the survival of neuronal progenitors and normal formation of the basal forebrain, striatum, thalamus and neocortex. Development.

[bib51] Metzger Marco, Bareiss Petra M., Danker Timm, Wagner Silvia, Hennenlotter Joerg, Guenther Elke, Obermayr Florian, Stenzl Arnulf, Koenigsrainer Alfred, Skutella Thomas, Just Lothar (2009). Expansion and differentiation of neural progenitors derived from the human adult enteric nervous system. Gastroenterology.

[bib52] Mullen R.J., Buck C.R., Smith A.M. (1992). NeuN, a neuronal specific nuclear protein in vertebrates.. Development.

[bib53] Osorio Nancy, Delmas Patrick, Jones Peter A. (2011). Patch clamp recording from enteric neurons in situ. Nat. Protoc..

[bib54] Paratore C., Goerich D.E., Suter U., Wegner M., Sommer L. (2001). Survival and glial fate acquisition of neural crest cells are regulated by an interplay between the transcription factor Sox10 and extrinsic combinatorial signaling. Development.

[bib55] Park D., Peng Xiang A., Fuxiang Mao F., Zhang L., Di C.-G., Liu X.-M., Shao Y., Ma B.-F., Lee J.-H., Ha K.-S., Walton N., Bruce T.L. (2010). Nestin is required for the proper self-renewal of neural stem cells. Stem Cells.

[bib56] Pozniak Christine D., Langseth Abraham J., Dijkgraaf Gerrit J.P., Choe Youngshik, Werb Zena, Pleasure Samuel J. (2010). Sox10 directs neural stem cells toward the oligodendrocyte lineage by decreasing Suppressor of Fused expression. Proc. Natl. Acad. Sci. USA.

[bib57] Progatzky F., Pachnis V. (2022). The role of enteric glia in intestinal immunity. Curr. Opin. Immunol..

[bib58] Ramoa A.S., McCormick D.A. (1994). Developmental changes in electrophysiological properties of LGNd neurons during reorganization of retinogeniculate connections. J. Neurosci..

[bib59] Reynolds B.A., Weiss S. (1996). Clonal and population analyses demonstrate that an EGF-responsive mammalian embryonic CNS precursor is a stem cell. Dev. Biol..

[bib60] Schäfer K.H., Ginneken C.V., Copray S. (2009). Plasticity and neural stem cells in the enteric nervous system. Anat. Rec. (Hoboken).

[bib61] Schäfer Karl-Herbert, Hagl Cornelia Irene, Rauch Ulrich (2003). Differentiation of neurospheres from the enteric nervous system. Pedia Surg. Int.

[bib62] Schonkeren Simone L., Küthe Tara T., Idris Musa, Bon-Frauches Ana C., Boesmans Werend, Melotte Veerle (2022). The gut brain in a dish: Murine primary enteric nervous system cell cultures. Neurogastroenterol. Motil..

[bib63] Schwaller Beat (2014). Calretinin: from a "simple" Ca(2+) buffer to a multifunctional protein implicated in many biological processes. Front Neuroanat..

[bib64] Seki Toshiyuki, Yanaihara Nozomu, Shapiro Jason Solomon, Saito Misato, Tabata Junya, Yokomizo Ryo, Noguchi Daito, Kuroda Takafumi, Kawabata Ayako, Suzuki Jiro, Takahashi Kazuaki, Matsuzawa Haruka, Miyake Misayo, Takenaka Masataka, Iida Yasushi, Yanagida Satoshi, Okamoto Aikou (2021). Interleukin-6 as an enhancer of anti-angiogenic therapy for ovarian clear cell carcinoma. Sci. Rep..

[bib65] Shen Qin, Goderie Susan K., Jin Li, Karanth Nithin, Sun Yu, Abramova Natalia, Vincent Peter, Pumiglia Kevin, Temple Sally (2004). Endothelial cells stimulate self-renewal and expand neurogenesis of neural stem cells. Science.

[bib66] Smith Tricia H., Ngwainmbi Joy, Grider John R., Dewey William L., Akbarali Hamid I. (2013). An in-vitro preparation of isolated enteric neurons and glia from the myenteric plexus of the adult mouse. J. Vis. Exp..

[bib67] Spencer Nick J., Hu Hongzhen (2020). Enteric nervous system: sensory transduction, neural circuits and gastrointestinal motility. Nat. Rev. Gastroenterol. Hepatol..

[bib68] Sun Jinqiao, Zhou Wenhao, Ma Duan, Yang Yi (2010). Endothelial cells promote neural stem cell proliferation and differentiation associated with VEGF activated Notch and Pten signaling. Dev. Dyn..

[bib69] Tewari Bhanu P., Chaunsali Lata, Campbell Susan L., Patel Dipan C., Goode Adam E., Sontheimer Harald (2018). Perineuronal nets decrease membrane capacitance of peritumoral fast spiking interneurons in a model of epilepsy. Nat. Commun..

[bib70] Tripathy Shreejoy J., Savitskaya Judith, Burton Shawn D., Urban Nathaniel N., Gerkin Richard C. (2014). NeuroElectro: a window to the world's neuron electrophysiology data. Front. Neuroinform.

[bib71] Uesaka Toshihiro, Nagashimada Mayumi, Enomoto Hideki (2013). GDNF signaling levels control migration and neuronal differentiation of enteric ganglion precursors. J. Neurosci..

[bib72] Villanacci V., Bassotti G., Nascimbeni R., Antonelli E., Cadei M., Fisogni S., Salerni B., Geboes K. (2008). Enteric nervous system abnormalities in inflammatory bowel diseases. Neurogastroenterol. Motil..

[bib73] Wahba G., Hebert A.-E., Grynspan D., Staines W., Schock S. (2016). A rapid and efficient method for dissociated cultures of mouse myenteric neurons. J. Neurosci. Methods.

[bib74] Wang Xiao-Yang, Yin Yuzhi, Yuan Hongyan, Sakamaki Toshiyuki, Okano Hideyuki, Glazer Robert I. (2008). Musashi1 modulates mammary progenitor cell expansion through proliferin-mediated activation of the Wnt and Notch pathways. Mol. Cell Biol..

[bib75] Xiong S., Puri P., Nemeth L., D S O′Briain, Reen D.J. (2000). Neuronal hypertrophy in acute appendicitis. Arch. Pathol. Lab Med.

[bib76] Young Kaylene M., Merson Tobias D., Sotthibundhu Areechun, Coulson Elizabeth J., Bartlett Perry F. (2007). p75 neurotrophin receptor expression defines a population of BDNF-responsive neurogenic precursor cells. J. Neurosci..

[bib77] Zhang Yonggang, Hu Wenhui (2013). Mouse enteric neuronal cell culture. Methods Mol. Biol..

[bib78] Zhang Yonggang, Hu Wenhui (2021). Mouse enteric neuronal cell culture. Methods Mol. Biol..

