# Supplementary material for: A novel method for culturing enteric neurons generates neurospheres containing functional myenteric neuronal subtypes
Source: J Neurosci Methods. Author manuscript; Available in PMC 2024 Jul 1. (PMC11144385; doi:10.1016/j.jneumeth.2024.110144)
Supplement: Supplemental Materials [file NIHMS1991190-supplement-Supplemental_Materials.docx]

**Supplemental Table: Material and reagent list with preferred manufacturer and catalog number.**

| **Name** | **Company** | **Calalog Number** |
| --- | --- | --- |
| BSA | MilliporeSigma | A7030 |
| DPBS | ThermoFisher Scientific | 14190144 |
| HBSS | ThermoFisher Scientific | 14025-092 |
| Advanced DMEM/F12 | ThermoFisher Scientific | 12634-010 |
| Pen-Strep | ThermoFisher Scientific | 15140122 |
| B27 plus supplement | ThermoFisher Scientific | A3582801 |
| N2 supplement | ThermoFisher Scientific | 17502048 |
| HEPES | ThermoFisher Scientific | 15630080 |
| Glutamax | ThermoFisher Scientific | 35050061 |
| FBS heat inactivated | ThermoFisher Scientific | 10082139; A3840001 |
| Steriflip-GP centrifuge tube top filter unit | MilliporeSigma | SCGP00525 |
| Sterile cotton tipped applicators | Fisher Scientific | 22029-504 |
| Silicone coated black petri dish | Living Systems Instrumentation | DD-90-S-BLK; DD-50-S-BLK |
| Reversible strainer 70 µm | Stemcell Technologies | 27260 |
| Reversible strainer 37 µm | Stemcell Technologies | 27250 |
| Matrigel GFR | Fisher Scientific | CB-40230C |
| Collagen, Type I | MilliporeSigma | C3867 |
| EGF protein CF | ThermoFisher Scientific | PMG8041 |
| FGF2 (bFGF) | R&D Systems | 3139-FB-025 |
| GDNF | R&D Systems | 212-GD-010 |
| Heparin solution, 0.2 % | StemCell Technologies | 07980 |
| Petri dishes 35 mm glass bottom | MatTek | P35G-1.5-14-C |
| Concavity slides | Fisher Scientific | 50-949-425 |
| PFA, 4% in PBS | Fisher Scientific | NC1266351 |
| Triton X-100 | MilliporeSigma | T8787 |
| Propidium iodide | ThermoFisher Scientific | P3566 |
| Normal goat serum | ThermoFisher Scientific | 10000C |
| Slow Fade DAPI | ThermoFisher Scientific | S36964 |
| TUBB3 | BioLegend | 801202 |
| GFAP | MilliporeSigma | MAB3402 |
| VE Cadherin | Abcam | ab205336 |
| ChAT | Abcam | ab181023 |
| Calretinin | ThermoFisher Scientific | MA5-14540 |
| nNOS | Enzo | ALX-210-529 |
| Sox2 | Abcam | ab92494 |
| Sox10 | Abcam | ab227680 |
| Msi1 | Abcam | ab52865 |
| Ki67 | Abcam | ab16667 |
| Nestin | ThermoFisher Scientific | MA1-110 |
| P75^NTR^ | Abcam | ab52987 |
| PGP9.5 | Abcam | ab108986 |
| HuD/C | Abcam | ab184267 |
| NeuN | Abcam | ab177487 |
| Tetrodotoxin |  |  |
| Lucifer yellow | MilliporeSigma | L0259 |
| Mouse IgG1 | BioLegend | 401402 |
| Mouse IgG2a | BioLegend | 401502 |
| Rabbit IgG | ThermoFisher Scientific | 02-6102 |
| Goat anti-mouse IgG F(ab)2 Alexa Fluor 488 | Jackson ImmunoResearch | 115-546-062 |
| Goat anti-mouse IgG F(ab)2 Alexa Fluor 594 | Jackson ImmunoResearch | 115-586-062 |
| Goat anti-rabbit IgG F(ab)2 Alexa Fluor 488 | Jackson ImmunoResearch | 111-546-144 |
| Goat anti-rabbit IgG F(ab)2 Alexa Fluor 594 | Jackson ImmunoResearch | 111-586-144 |
| Liberase TH | MilliporeSigma | 05401135001 |
| DNase I | MilliporeSigma | 4716728001 |
